# Prevalence and Characterization of *Salmonella* Isolated from Chickens in Anhui, China

**DOI:** 10.3390/pathogens12030465

**Published:** 2023-03-16

**Authors:** Xuehuai Shen, Lei Yin, Anyun Zhang, Ruihong Zhao, Dongdong Yin, Jieru Wang, Yin Dai, Hongyan Hou, Xiaocheng Pan, Xiaomiao Hu, Danjun Zhang, Yongjie Liu

**Affiliations:** 1 College of Veterinary Medicine, Nanjing Agricultural University, Nanjing 210095, China; 2 Anhui Province Key Laboratory of Livestock and Poultry Product Safety Engineering, Livestock and Poultry Epidemic Diseases Research Center of Anhui Province, Institute of Animal Husbandry and Veterinary Science, Anhui Academy of Agricultural Science, Hefei 230001, China; 3 Key Laboratory of Bio-Resource and Eco-Environment of Ministry of Education, Animal Disease Prevention and Food Safety Key Laboratory of Sichuan Province, College of Life Sciences, Sichuan University, Chengdu 610017, China

**Keywords:** *Salmonella*, chicken, serovar, antimicrobial susceptibility, antibiotic resistance genes, biofilm formation, virulence genes, multilocus sequence typing

## Abstract

*Salmonella* is one of the most important zoonotic pathogens that can cause both acute and chronic illnesses in poultry flocks, and can also be transmitted to humans from infected poultry. The purpose of this study was to investigate the prevalence, antimicrobial resistance, and molecular characteristics of *Salmonella* isolated from diseased and clinically healthy chickens in Anhui, China. In total, 108 *Salmonella* isolates (5.66%) were successfully recovered from chicken samples (*n* = 1908), including pathological tissue (57/408, 13.97%) and cloacal swabs (51/1500, 3.40%), and *S.* Enteritidis (43.52%), *S.* Typhimurium (23.15%), and *S.* Pullorum (10.19%) were the three most prevalent isolates. *Salmonella* isolates showed high rates of resistance to penicillin (61.11%), tetracyclines (47.22% to tetracycline and 45.37% to doxycycline), and sulfonamides (48.89%), and all isolates were susceptible to imipenem and polymyxin B. In total, 43.52% isolates were multidrug-resistant and had complex antimicrobial resistance patterns. The majority of isolates harbored *cat1* (77.78%), *blaTEM* (61.11%), and *blaCMY-2* (63.89%) genes, and the antimicrobial resistance genes in the isolates were significantly positively correlated with their corresponding resistance phenotype. *Salmonella* isolates carry high rates of virulence genes, with some of these reaching 100% (*invA*, *mgtC*, and *stn*). Fifty-seven isolates (52.78%) were biofilm-producing. The 108 isolates were classified into 12 sequence types (STs), whereby ST11 (43.51%) was the most prevalent, followed by ST19 (20.37%) and ST92 (13.89%). In conclusion, *Salmonella* infection in chicken flocks is still serious in Anhui Province, and not only causes disease in chickens but might also pose a threat to public health security.

## 1. Introduction

*Salmonella enterica* is one of the most frequent zoonotic pathogens causing human and animal infections worldwide, comprising a wide variety of serovars, with over 2600 identified [1]. *Salmonella* is commonly found in both domestic and wild animals, including poultry, pigs, and cattle. Poultry products in particular have been identified as a significant source of human salmonellosis [2]. Foodborne salmonellosis is the most relevant source, with a high global impact on human health, although there are other sources, such as animal/reptile, environmental, or human-to-human sources [3]. Compared with other foodborne microorganisms, *Salmonella* is the most common cause of hospitalization and death [4]. According to 2018 data, nontyphoid *Salmonella* infection caused approximately 33 million human deaths worldwide. Although there are various serovars linked to salmonellosis, only a few are accountable for the majority of human infections. The primary serovars responsible for human infections in the EU and USA are *S*. Enteritidis and *S*. Typhimurium [5,6].

The identification of serovars is essential for epidemiological surveillance and disease assessment, as various serovars of *Salmonella* exhibit distinct host ranges and disease-causing capabilities [7]. Some serovars, for example, *S.* Enteritidis and *S.* Typhimurium, can infect not only poultry but also humans [8]. By contrast, *S.* Pullorum and *S.* Gallinarum only induce illness in chickens, causing pullorum disease and fowl typhoid, respectively, leading to considerable economic losses in the poultry industry [9,10]. Therefore, chickens are a prominent source of infection and act as a reservoir for *Salmonella*. Xu et al. [11] identified 12 serovars of *Salmonella* from dead embryo samples collected from breeder chicken hatcheries in Henan, China. The dominant serovar was *S.* Pullorum (75.79%), followed by *S.* Enteritidis (7.14%). In Zhao et al.’s study [12] in Shandong, China, *S.* Thompson (37.20%) and *S.* Infantis (32.60%) were the most prevalent isolates from dead-in-shell chicken embryos. The majority of isolates (66.30%) were resistant to ampicillin, while 55.80% of isolates exhibited multidrug resistance (MDR). Chicken embryos and eggs infected with *Salmonella* can not only cause the vertical transmission of these bacteria during hatching, but they are also an important cause of human foodborne infection [13]. Another study showed that the most common serovars were *S.* Kentucky (44.7%) and *S.* Enteritidis (32.5%) at different chicken-slaughtering stages using whole-genome sequencing in Jiangsu, China [14]. In large-scale breeder farms, *S.* Enteritidis was found to be the most common serovar, with high rates of antimicrobial resistance to nalidixic acid (100.0%), streptomycin (100.0%), ampicillin (98.4%), and erythromycin (93.7%) [15]. An investigation by Wang et al. on *Salmonella* contamination of retail meats in Anhui Province markets found that *S.* Enteritidis and *S.* Typhimurium were the most prevalent serovars, with high resistance rates to ampicillin (87.5%), doxycycline (75.0%), and tetracycline (62.5%) [16].

The results of these previous studies provided strong data support for *Salmonella* infection in poultry, allowing for the prevention and control of its human foodborne infection. However, there have been few studies regarding the prevalence and characteristics of *Salmonella* isolated from chickens in Anhui Province [17]. The aim of the present work is to study the serovars’ prevalence, phenotypic antimicrobial resistance profile, and molecular characteristics of *Salmonella* isolated from diseased and clinically healthy chickens in Anhui Province to better understand the epidemiology of *Salmonella* in chickens in Anhui, China, and to provide a research basis for the prevention and control of this foodborne pathogen.

## 2. Materials and Methods

### 2.1. Sample Collection and Culture of Salmonella

From March 2019 to April 2022, 408 pathological tissue samples (liver, spleen, and kidneys) were collected at the Anhui Academy of Agricultural Sciences’ Veterinary Clinical Diagnosis Guidance Center. These pathological samples were obtained from diseased chickens with bacterial infections in layer and broiler farms, some of which exhibited the histopathological changes suspected of *Salmonella* infection, such as enlarged liver and spleen, small necrotic spots, and copper-green lesions in the liver. In addition, we collected 1500 cloacal swab samples from clinically healthy chickens from 50 chicken farms (30 cloacal swab samples/farm), including 45 layer farms and 5 broiler farms (19 large-scale, 28 medium-scale, and 3 small-scale). The prevalence of cloacal swab samples is shown in Table S1. This work was a monitoring task of the National Animal Disease Data Center. All samples came from 16 cities in Anhui province: Anqing, Changfeng, Chaohu, Dingyuan, Fanchang, Feidong, Fuyang, Feixi, Guzhen, Hefei, Hexian, Huainan, Huoqiu, Lu’an, Shouxian, and Wuhu.

*Salmonella* isolates were isolated and identified using previously reported methods [12]. To summarize, 10 g of pathological tissue (mixed sample containing multiple pathological tissues from the same source) or cloacal swab sample was added with 100 mL of buffered peptone water (BPW; Hopebiol, Qingdao, China) and incubated at 37 °C for 12 h. Then, 1 mL of enriched BPW suspension was transferred to 10 mL of selenite cysteine (SC; Hopebiol) at 42 °C for 24 h, which was further streaked on xylose lysine tergitol 4 (XLT4; Hopebiol) agar plates and incubated at 37 °C for 24 h for *Salmonella* selection. Then, the isolates were subjected to DNA extraction using a bacterial genome extraction kit (Beijing Solarbio Science Technology Co., Ltd., Beijing, China). PCR was performed to amplify the 16S rRNA [18]. The obtained amplicons were sequenced and then aligned using the NCBI database https://blast.ncbi.nlm.nih.gov/Blast.cgi (accessed on 8 June 2022).

### 2.2. Serotyping of Salmonella Isolates

The *Salmonella* isolates were subcultured and serotyped using commercial O and H antisera through slide agglutination, following the manufacturer’s guidelines (Tianrun Bio Pharmaceutical, Ningbo, China). All identified *Salmonella* isolates were added to the lyophilization protectant and stored at −80 °C for further use.

### 2.3. Antimicrobial Susceptibility Testing

*Salmonella* sensitivity to 24 different common antimicrobials from 14 classes of antimicrobials (Hangzhou microbial reagent Co., Ltd., Hangzhou, China) was evaluated using a Kirby–Bauer disk diffusion approach, following the protocols of the Clinical and Laboratory Standards Institute (CLSI) [19]. The selected antimicrobials are frequently utilized to manage bacterial infections in animals and humans. The antimicrobials used for these tests comprised ampicillin (AMP), amoxicillin/clavulanic acid (AMC), ceftriaxone (CRO), cefotaxime (CTX), cephalexin (CN), gentamicin (GEN), amikacin (AMK), neomycin (NEO), tetracycline (TET), doxycycline (DOX), ciprofloxacin (CIP), enrofloxacin (ENR), levofloxacin (LEV), norfloxacin (NOR), chloramphenicol (CHL), florfenicol (FLO), sulfamethoxazole (SXT), trimethoprim (TMP), azithromycin (AZM), furazolidone (FUR), imipenem (IPM), polymyxin B (PB), fosfomycin (FOS), and aztreonam (AZT). The ATCC 25,922 *Escherichia coli* strain was used as the quality control (Hopebiol). All *Salmonella* isolates that were found to be resistant to more than three antimicrobials classes were defined as being MDR isolates.

### 2.4. Prevalence of Drug Resistance and Virulence Genes in Salmonella Isolates

Briefly, all *Salmonella* isolates preserved at −80 °C were streaked onto tryptic soy agar and incubated at 37 °C for 12 h. Subsequently, one colony was selected, inoculated into BPW, and incubated at 37 °C overnight. The next day, 1 mL of bacterial solution was collected and centrifuged at 5000× *g* for 5 min, and then commercial kits (Beijing Solarbio Science Technology Co., Ltd., Beijing, China) were used to extract DNA from the isolates. We used conventional PCR methods to identify 14 drug resistance genes and 7 virulence genes in all the isolates. Resistance genes included *blaTEM*, *blaCMY-2*, *aadA1*, *strA*, *aph(3′)-IIa*, *aac(6′)-Ib-cr*, *qnrB*, *qnrS*, *sul1*, *sul2*, *tetA*, *tetB*, *cat1*, and *floR*. Virulence genes included *invA*, *sseL*, *mgtC*, *siiE*, *sopB*, *spvB*, and *stn*. The sequences of the PCR primers for all the genes are shown in Table 1. The primers were obtained from Anhui General Biotechnology Co., Ltd. (Anhui, China), and the PCR reaction was conducted with a Takara Premix Taq kit (Takara Bio Inc., Dalian, China) in a total volume of 20 µL. PCR products were identified using a gel imaging system after treatment with GoldView nucleic acid stain (Beijing Solarbio Science Technology Co., Ltd., Beijing, China).

### 2.5. Biofilm Assay

The amount of biofilm formed by *Salmonella* isolates was measured using the 96-well polystyrene microtiter plate test method described by Yin et al. [24]. *Pseudomonas aeruginosa* ATCC 27,853 (Hopebiol, Qingdao, China) was utilized as a positive control, while negative control wells were filled with 200 µL of TSB only. Six replicate wells were used to test each isolate as well as the negative control wells. The amount of biofilm formed by each tested isolate was determined by calculating the average optical density at 570 nm (OD570) using the absorbance of six wells measured by INFINITE 200PRO (Tecan Austria GmbH, Grödig, Austria). The biofilm-forming abilities of the *Salmonella* isolates were classified into four groups based on a comparison of the optical density (OD) of the test wells and the negative control wells (ODc): (i) no biofilm producer: OD ≤ ODc; (ii) weak biofilm producer: ODc < OD ≤ (2 × ODc); (iii) moderate biofilm producer: (2 × ODc) < OD ≤ (4 × ODc); and (iv) strong biofilm producer: (4 × ODc) < OD.

### 2.6. Multilocus Sequence Typing (MLST)

The *Salmonella* isolates were analyzed by MLST, which involved amplifying seven housekeeping genes (*aroC*, *dnaN*, *hemD*, *hisD*, *purE*, *sucA*, and *thrA*) according to previously described protocols [25]. PCR amplifications were conducted in a 25 µL volume containing 12.5 µL of 2 × Taq PCR Mix (Takara Bio Inc.), 2 µL of template, 1 µL of each 20 µM primer, and 8.5 µL of sterile ddH2O. The conditions for PCR reactions were obtained from the *Salmonella* MLST website and database http://mlst.warwick.ac.uk/mlst/dbs/Senterica/ (accessed on 25 August 2022). The PCR samples were purified using gel electrophoresis and sent for bidirectional DNA sequencing at Anhui General Biotechnology Co., Ltd. Each gene sequence was submitted to the *Salmonella* MLST database for comparison to obtain the specific *Salmonella* sequence type. The MUSCLE alignment program was utilized to align all sequences [26], and a phylogenetic tree was constructed using the neighbor-joining method in MEGA 7.0 [27].

### 2.7. Statistical Analysis

All data were preliminarily processed using Excel 2010 (Microsoft, Redmond, WA, USA). The *Salmonella* serovar distribution, cluster heat map, and correlation analysis were drawn using Excel 2010 and GraphPad Prism 7 software (GraphPad Inc., La Jolla, CA, USA), respectively. The determination results of the *Salmonella* isolates’ biofilm-forming abilities were processed by Excel 2010 (Microsoft, Redmond, WA, USA) and the experiments were repeated three times.

## 3. Results

### 3.1. Isolation and Serotyping of Salmonella

**Figure 1:**
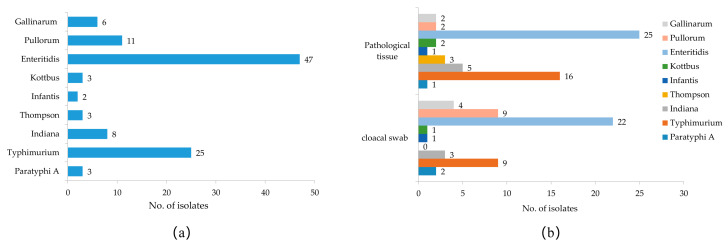
Serovar prevalence of *Salmonella* isolates isolated from chickens in Anhui. (**a**) Serovar distribution of all *Salmonella* isolates. (**b**) Serovar distribution of *Salmonella* from different sample sources.

In this study, 1908 samples were collected, including 1500 cloacal swab samples and 408 pathological tissue samples. In total, 108 strains of *Salmonella* were isolated and identified from all the samples by isolation culture and PCR 16S rRNA amplification sequencing, with an overall *salmonella* isolation rate of 5.66% (Table 2). Among them, 51 isolates were from cloacal swab samples, with an isolation rate of 3.40%, and 57 isolates were from pathological tissue samples, with an isolation rate of 13.97%. The pathological tissue samples had a higher *Salmonella* isolation rate than the cloacal swab samples, and the prevalence information for all *Salmonella* isolates is shown in Table S2. A total of 9 different serovars were identified among the 108 *Salmonella* isolates, and *S.* Enteritidis (43.52%), *S.* Typhimurium (23.15%), and *S.* Pullorum (10.19%) were the most frequent serovars in the chicken samples (Figure 1a). The prevalence of *Salmonella* serovars in different samples varied; for example, the proportions of *S.* Pullorum (9/51, 17.65%) and *S.* Gallinarum (4/51, 7.84%) in cloacal swab samples were higher than in the pathological tissue samples (both 2/57, 3.51%), and *S.* Thompson was isolated only from tissue samples from diseased chickens (Figure 1b).

### 3.2. Antibiotic Susceptibility Testing

**Figure 2:**
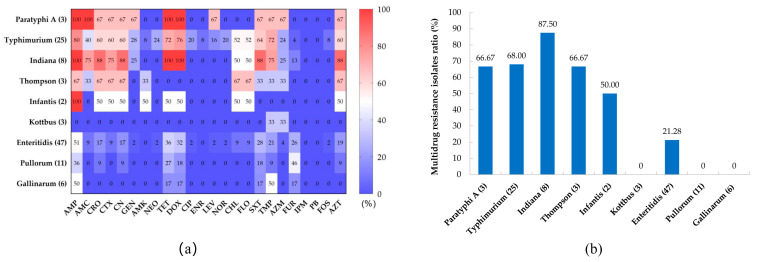
The prevalence of antimicrobial resistance among different serovars of *Salmonella*. (**a**) Heat map of antibiotic resistance distribution among different serovars of *Salmonella*. (**b**) Distribution of multidrug-resistant isolates among different serovars of *Salmonella*.

The susceptibilities of the 108 *Salmonella* isolatesfrom chickens to 24 antibiotics are shown in Table 3. The results revealed high rates of resistance to ampicillin (61.11%), tetracycline (47.22%), doxycycline (45.37%), sulfamethoxazole (48.89%), trimethoprim (48.89%), and aztreonam (34.26%). About 30% of *Salmonella* isolates were resistant to cephems, namely ceftriaxone (33.33%), cefotaxime (27.78%), and cephalexin (33.33%); and 22.22% of *Salmonella* isolates were resistant to chloramphenicols (chloramphenicol and florfenicol). *Salmonella* isolates showed low resistance rates to aminoglycoside, quinolone, and macrolide antibiotics; however, all isolates were susceptible to imipenem and polymyxin B. Regarding serovars (Figure 2a), the antibiotic resistance rates of *S.* Paratyphi A, *S.* Typhimurium, *S.* Indiana, and *S.* Thompson were higher than those of *S.* Kottbus, *S.* Enteritidis, *S.* Pullorum, and *S.* Gallinarum.

The antimicrobial resistance spectrum of the isolates is shown in Table S3. Chicken-derived *Salmonella* isolates in Anhui have a complex antibiotic resistance spectrum, among which 43.52% (47 isolates) were MDR isolates; however, 18.52% (20 isolates) were still sensitive to all antibiotics. Strikingly, one *S.* Typhimurium isolate was resistant to 20 antibiotics. The proportion of MDR isolates among *S.* Indiana isolates was the highest, at 87.50%, followed by *S.* Typhimurium (68.00%), *S.* Thompson (66.67%), and *S.* Paratyphi A (66.67%). Only 21.28% of *S.* Enteritidis isolates were MDR isolates, while no MDR isolates appeared among *S.* Pullorum, *S.* Gallinarum, and *S.* Kottbus isolates (Figure 2b). There is a certain correlation between the resistance phenotypes of isolates to different classes of antimicrobials (Figure S1). The resistance phenotypes of aminoglycosides and quinolone antimicrobials show a significantly positive correlation, as well as a positive correlation between tetracyclines and chloramphenicol-resistant phenotypes. In total, 95.83% (23/24) of chloramphenicol-resistant *Salmonella* isolates exhibited co-resistance to chloramphenicol and cefotaxime, and all ciprofloxacin-resistant isolates were resistant to both gentamicin and neomycin.

### 3.3. Prevalence of Antimicrobial-Resistance-Related Genes in Salmonella Isolates

**Figure 3:**
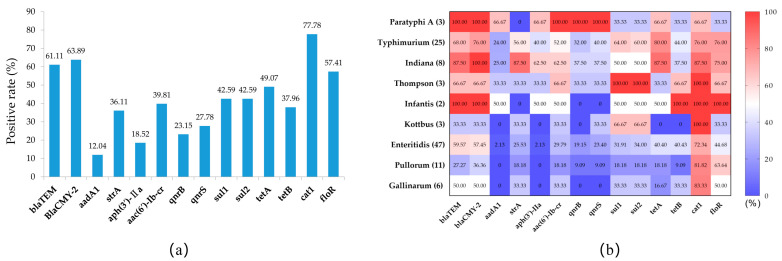
The prevalence of antimicrobial resistance genes in *Salmonella* isolates. (**a**) Prevalence of antimicrobial resistance genes in all *Salmonella* isolates. (**b**) Heat map of the prevalence of antimicrobial resistance genes among different *Salmonella* serovars.

The results of the PCR analysis for antimicrobial resistance genes are shown in Figure 3a. The carrier rates of the two β-lactamase genes, *blaTEM* and *blaCMY-2*, were 61.11% and 63.89%, respectively; the carrier rates of aminoglycoside-resistance-related genes *aadA*, *strA*, and *aph(3′)-IIa* were 12.40%, 36.11%, and 18.52%, respectively; the carrier rates of quinolone-resistance-related genes *aac(6′)-Ib-cr*, *qnrB*, and *qnrS* were 39.81%, 23.15% and 27.78%, respectively; the carrier rates of sulfonamide-resistance-related genes *sul1* and *sul2* were both 42.59%; and the carrier rates of the tetracycline resistance genes *tetA* and *tetB* were 49.07% and 37.96%, respectively. All isolates had high carrying rates of chloramphenicol-resistance-related genes, with 77.78% (*catA1*) and 54.71% (*floR)*, respectively.

There were some differences in the carrier rates of drug-resistance-related genes among different serovars (Figure 3b). Overall, the carrier rate of the drug-resistance-related genes of *S.* Paratyphi A, *S.* Typhimurium, *S.* Indiana, *S.* Thompson, and *S.* Infantis were higher than those of *S.* Enteritidis, *S.* Pullorum, and *S.* Gallinarum. However, the chloramphenicol-resistance-related genes *cat1* and *floR* had higher positive rates in *S.* Enteritidis, *S.* Pullorum, and *S.* Gallinarum than in the other serovars. Furthermore, these resistance-related genes showed higher carrier rates in isolates with corresponding resistance phenotypes (Table 4), e.g., the carrier rates of *aac(6’) Ib-cr*, *qnrB*, and *qnrS* were 100% in quinolone-resistant isolates, while the carrier rates *aadA* and *aph(3′)-IIa* were both 93.33% in isolates that were resistant to aminoglycoside antibiotics.

**Figure 4:**
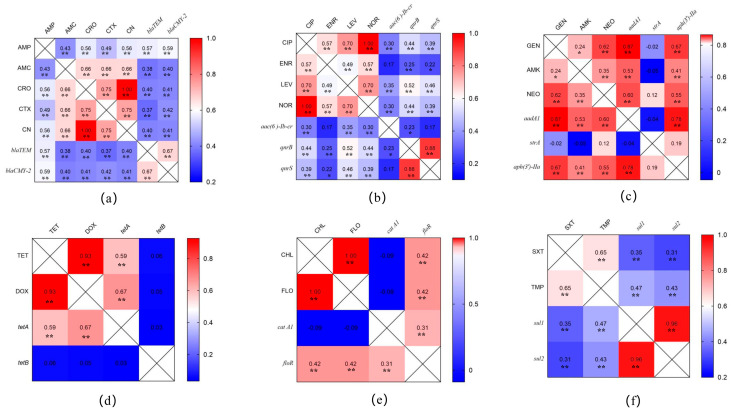
Correlation analysis between the antimicrobial-resistance-phenotype- and their resistance−gene−carrying *Salmonella* isolates. (**a**) Correlation analysis of β-lactamase−resistance−genes with their resistance to penicillins and cephalosporins. (**b**) Correlation analysis of quinolone−resistance−genes with their resistance to quinolones. (**c**) Correlation analysis of aminoglycoside−resistance−genes with their resistance to aminoglycosides. (**d**) Correlation analysis of tetracycline−resistance−genes with their resistance to tetracyclines. (**e**) Correlation analysis of chloramphenicol−resistance−genes with their resistance to chloramphenicols. (**f**) Correlation analysis of sulfonamide−resistance−genes with their resistance to sulfonamides. * *p* < 0.05, ** *p* < 0.01, correlation analysis by pearson method.

The results of the correlation analysis between the drug-resistance-phenotype- and drug- resistance-gene-carrying *Salmonella* isolates are shown in Figure 4. The carrier rates of β-lactamase genes (*blaTEM* and *blaCMY-2*) in isolates are significantly correlated with their resistance to penicillin and cephalosporins (Figure 4a). The carrier rates of quinolone (*aac(6’) Ib-cr*, *qnrB*, and *qnrS*)- and sulfonamide (*sul1* and *sul2*)-resistance-related genes in isolates are significantly correlated with their resistance to quinolones and sulfonamide antimicrobials (Figure 4b,f). However, some aminoglycoside (Figure 4c)-, tetracycline (Figure 4d)- and chloramphenicol-resistance-related genes (Figure 4e) carried in isolates were not correlated to their resistance phenotypes, such as *strA*, *tetB*, and *cat A1*.

### 3.4. The Prevalence of Virulence Genes and the Biofilm-Producing Ability of Salmonella Isolates

**Figure 5:**
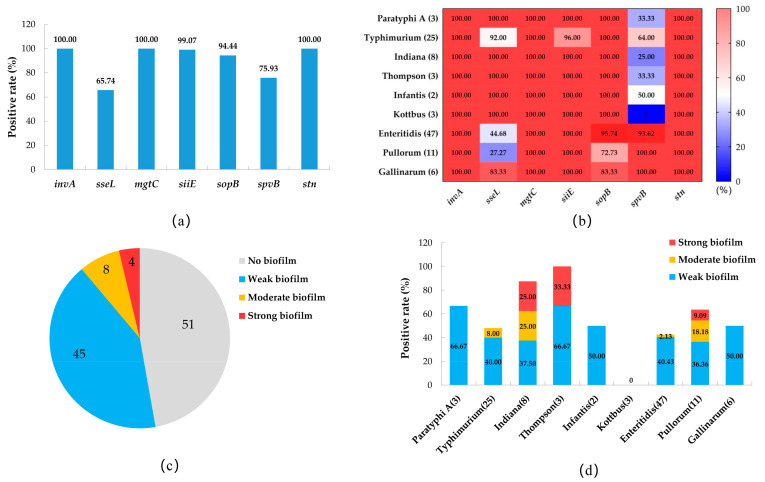
Prevalence of virulence genes and characteristics of biofilm-producing *Salmonella* isolates. (**a**) Prevalence of virulence genes of all *Salmonella* isolates. (**b**) Heat map of the prevalence of virulence genes among different serovars of *Salmonella* isolates. (**c**) Number of *Salmonella* isolates with different biofilm-producing abilities. (**d**) Prevalence of isolates with different biofilm-producing abilities among different *Salmonella* serovars.

The prevalence of virulence genes in the *Salmonella* isolates is shown in Figure 5a. The virulence genes *invA*, *mgtC*, and *stn* were present in all isolates. The carrier rates of *siiE*, *sopB*, and *spvB* were 99.07%, 94.44%, and 75.93, respectively, whereas that of the *sseL* gene was lower, at 65.2%. In the serovars, the positive carrier rates of *sseL* and *sopB* genes in *S.* Enteritidis, *S.* Pullorum, and *S.* Gallinarum isolates were lower than those in the other serovars, while the prevalence of the *spvB* gene in different serovars showed the opposite pattern (Figure 5b).

The biofilm assay results of the *Salmonella* isolates showed that 57 isolates (52.78%) had the ability to form biofilms, among which 45 isolates (41.67%) were a weak biofilm producer, 8 isolates (7.41%) were a moderate biofilm producer, and 4 isolates (3.70%) were a strong biofilm producer (Figure 5c). Among the serovars, all serovars of *Salmonella* except *S.* Kottbus had biofilm-producing isolates, among which *S.* Thompson had a higher proportion (3/3, 100%), followed by *S.* Indiana (7/8, 87.50%), *S.* Paratyphi A (2/3, 66.67%), and *S.* Pullorum (7/11, 63.64%). Three serovars (*S.* Paratyphi A, *S.* Infantis, and *S.* Gallinarum) only had weak biofilm-producing isolates, and the moderate and strong biofilm-producing isolates appeared in *S.* Typhimurium (two moderate isolates), *S.* Thompson (one strong and two moderate isolates), *S.* Indiana (two strong and two moderate isolates), and *S.* Pullorum (one strong and two moderate isolates) (Figure 5d).

### 3.5. MLST Analysis

**Figure 6:**
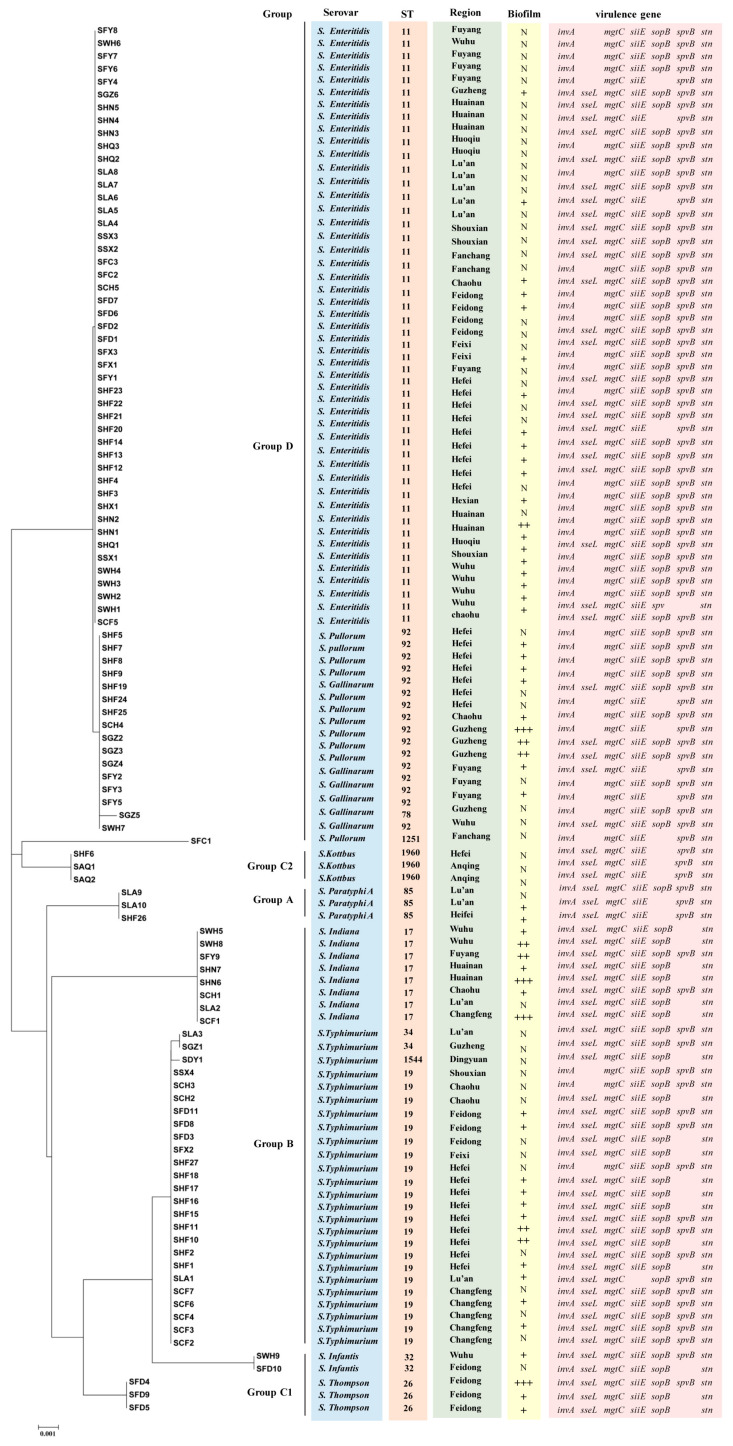
Phylogenetic tree of *Salmonella* isolate-based multilocus sequence typing. Markers include serovar group, serovars, ST-types, region, biofilm (“N” represent non-biofilm producer, “+” represent weak biofilm producer, “++” represent moderate biofilm producer, and “+++” represent strong biofilm producer), and virulence genes (*invA*, *sseL*, *mgtC*, *siiE, sopB, spvB* and *stn*; blank indicates that gene detection was negative) of *Salmonella* isolates.

**Figure 7:**
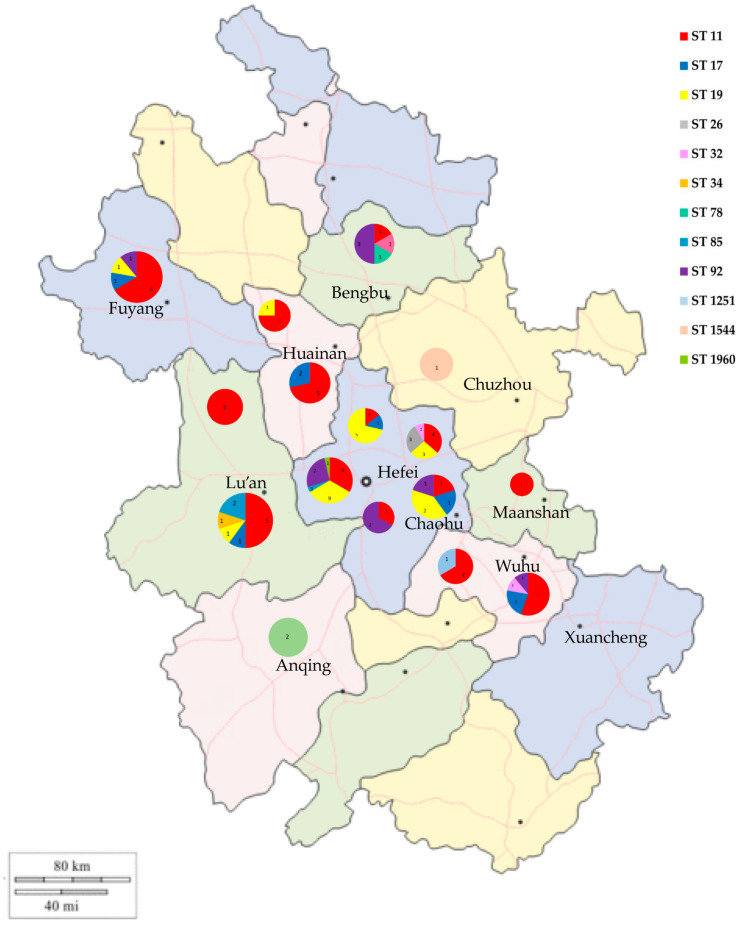
Geographical distribution of ST-type *Salmonella* isolates in Anhui Province.

In the MLST analysis (Figure 6), the 108 *Salmonella* isolates were classified into 12 ST types: ST11, ST17, ST19, ST26, ST32, ST34, ST78, ST85, ST92, ST1251, ST1544, and ST1960. ST11 was the most common ST in this study (47/108, 43.51%), followed by ST19 (20.37%), ST92 (13.89%), and ST17 (7.41%). ST11 had a wide distribution in several regions of Anhui and was the dominant ST type in Lu’an, Huainan, Fuyang, and Wuhu (Figure 7). ST19 was the most prevalent ST in *S. typhimurium* isolates (23/25, 92.00%), and was widely distributed in Changfeng, Hefei, Feidong, and Chaohu. ST92 isolates were mainly from Hefei, Guzhen, and Fuyang, and most of the isolates were isolated from cloacal swab samples of clinically healthy chickens. Furthermore, we observed that the majority of isolates sharing the same sequence types (STs) were also of the same serovars. For instance, strains with ST11 were identified as *S.* Enteritidis, while ST19, ST34, and 1544 were associated with *S.* Typhimurium, ST17 with *S.* Indiana, and ST1960 with *S.* Kottbus. However, ST92 corresponded to two serovars: *S.* Pullorum and *S.* Gallinarum.

## 4. Discussion

In this study, a total of 108 *Salmonella* isolates were identified from pathological tissue samples from diseased chickens and cloacal swab samples from clinically healthy chickens collected in 16 cities in Anhui. The overall isolation rate of *Salmonella* was 5.66%, similar to that reported by Zhao et al. [12] and Li et al. [28] from chicken farms and hatchery samples in Shandong and Shaanxi, respectively. However, it was lower than the isolation rates reported from commercial chicken farms in Henan [11] and Qingdao [29], China. The *Salmonella* isolation rate from clinically diseased chickens in the present study was higher than that reported by Wang et al. [25] in a clinical investigation of diseased chickens in north China. In any case, the isolation rate of *Salmonella* in slaughterhouses and chicken products was higher than in farms, which is an important *Salmonella* link between chicken production and food [30]. The variations in *Salmonella* isolation rates could be due to differences in region or season, or differences in the sampling techniques used across studies [31]. The poultry farming industry in Anhui Province is large, and according to statistics, there was an average annual stock of 70–80 million egg-laying hens and an average annual slaughter of 180–200 million broiler chickens in 2020–2022 (unofficial data). In this study, samples from laying and broiler farms with different breeding modes and scales in 16 cities in Anhui Province were used for an epidemiological study of *Salmonella* infection in chickens, and the results were representative. However, the sample isolation rates of *Salmonella* indicate that the *Salmonella* infection situation in chickens in Anhui remains serious, leading to the clinical morbidity of chickens, and more importantly, an invisible infection of *Salmonella* in clinically healthy chickens, which is a considerable mediator of horizontal and vertical *Salmonella* transmission in chickens.

Serotyping can be used as an effective method to assess the means of transmission and develop strategies for preventing the spread of disease within poultry facilities. [32]. Among the isolates, we identified nine serovars, of which *S.* Enteritidis was the most frequent, followed by *S.* Typhimurium and *S.* Pullorum. This is consistent with studies of commercial chicken farms in Shanghai and Sichuan [33,34], while *S.* Gallinarum-pullorum was found to be dominant in Henan [11]. Another study found that *S.* Enteritidis and *S.* Typhimurium were the most common serovars isolated from diseased poultry in northern China [25]. *S.* Enteritidis and *S.* Typhimurium are the most common serovars of *Salmonella* that cause human infection, resulting in severe gastrointestinal disease [35,36,37]. According to the China National Foodborne Diseases Surveillance Network, over the past decade (2010–2019), the most prevalent serovars in nontyphoidal *Salmonella* infections in Zhejiang Province were *S.* Enteritidis and *S.* Typhimurium [13]. Our findings suggested that the widespread distribution of foodborne *Salmonella* serovars in chicken flocks might pose a threat to food safety, and this conclusion was also confirmed by the literature [38,39]. Differences in the distribution of *Salmonella* serovars in different studies are related to regional differences and, in addition, might be related to the source and type of samples selected. For example, *S.* Pullorum and *S.* Enteritidis were the most common *Salmonella* serovars isolated from dead chicken embryos [11,25], while the more common serovars found in the slaughterhouse and chicken meat samples were *S.* Indiana [40], *S.* Typhimurium [41], or *S.* Enteritidis [42]. In the present study, the isolation rates of *S.* Pullorum and *S.* Gallinarum were higher in cloacal swabs than in pathological tissue samples. Although adult chickens infected with *S.* Pullorum may appear asymptomatic, the bacteria can persist for several months in the spleen and reproductive tract, leading to vertical transmission to eggs and progeny [43], which might also explain the high isolation rate of *S.* Pullorum in dead chicken embryos. However, the elimination of *S.* Pullorum has been carried out in many large breeder farms in China, and remarkable results have been achieved.

Animals are administered antimicrobials for various purposes, such as disease treatment, prevention, control, and growth/feed efficiency promotion [44]. Resistance to antibiotics has emerged as a significant global public health concern, with reports of bacteria isolated from animals displaying resistance to different antibiotics [45]. Despite efforts to limit antibiotic use in animal feeding, this study found that *Salmonella* isolates exhibited high rates of resistance to ampicillin, tetracycline, doxycycline, sulfamethoxazole, and trimethoprim. These resistance rates agree with reports on poultry *Salmonella* isolates in northern China [25], Shandong [12], and Guangdong [46], but are generally lower than those of Henan [11]. There were low resistance rates to aminoglycoside, quinolone, and macrolide antibiotics and no resistance to imipenem and polymyxin B, which contrasted with Zhao et al.’s report [7], in which the *Salmonella* isolates isolated from broiler chickens were 100% resistant to polymyxin B. These high resistance rates in *Salmonella* isolated from chickens might be attributed to the widespread use of antibiotics for animal breeding and disease control [47]. In addition, different serovars of *Salmonella* showed different antibiotic resistance patterns in our study, such as the antibiotic resistance rates of *S.* Typhimurium and *S.* Indiana, which were higher than those of *S.* Enteritidis and *S.* Pullorum. While certain serovars may not be currently dominant, their prevalence may shift over time, and they could potentially become the primary serovars in a given region due to exposure to multiple antimicrobial selection pressures [48].

In this study, a high prevalence of MDR patterns among the *Salmonella* isolates was detected, and 43.52% of *Salmonella* isolates presented resistance to more than three antibiotic classes, which was lower than that previously reported in Shandong (53.7%), Henan (69.64%), Guangdong (59.5%), and northern China (69.64%). Yang et al. [49] also showed that 86.7% of *Salmonella* isolates from Shanghai exhibited an MDR phenotype. There are some differences in the prevalence of MDR isolates in different serovars of *Salmonella*. In addition, *S.* Indiana showed the highest proportion of MDR isolates (87.50%), which was consistent with Zhang et al.’s report [50]. However, one study found different results from ours, namely that *S.* Enteritidis showed a high MDR rate [7]. We also found a certain correlation between the resistance phenotypes of different types of antibiotics among MDR isolates, such as 95.83% (23/24) of chloramphenicol-resistant *Salmonella* isolates exhibiting co-resistance to chloramphenicol and cefotaxime. According to Abd El-Aziz et al. [51], there are many XDR *Salmonella* isolates in livestock animals exhibiting co-resistance to ciprofloxacin (CIP) and tigecycline (TIG), and this co-resistance is facilitated by the overexpression of acrAB, which enhances efflux-mediated resistance to CIP/TIG. The phenomenon of co-resistance is prevalent in MDR pathogenic bacteria isolated from animals, such as amoxicillin and tetracycline co-resistance in *Escherichia coli* [52], and co-resistance to macrolides and fluoroquinolones in *Campylobacter* [53]. Therefore, the molecular mechanism of co-resistance remains to be further studied. Our findings indicated that it is necessary to continue monitoring antibacterial agents using them prudently in clinically, veterinary, and agricultural settings to avoid the development of cross-resistance.

The presence of antibiotic resistance genes (ARGs) is the origin and molecular basis of bacterial resistance [54]. The abundance of ARGs showed a significant statistical effect with antibiotic pressure, even at very low levels [55]. The extended-spectrum β lactamases are bacterial enzymes capable of hydrolyzing extended-spectrum cephalosporins and rendering beta-lactam antibiotics ineffective [56]. In this study, two β-lactamase genes, *blaTEM* and *blaCMY-2*, were found in 61.11% and 63.89% of the *Salmonella* isolates, respectively, which was similar to the proportions detected in *Salmonella* isolated from Shandong chicken flocks by Zhao et al. [12] and Alam et al. [8], indicating the high proportion of *Salmonella* isolates carrying β-lactamase genes in different cities or provinces. We found that the chloramphenicol-resistance-related genes *cat1* and *floR* were very common among the isolates. Florfenicol is a commonly used antibiotic in veterinary medicine; therefore, the emergence of these resistance genes might be related to the long-term use of this antibiotic [57]. Hence, further attention should be paid to the changes in the resistance of *Salmonella* to florfenicol. The rate of carrying aminoglycoside- and quinolone-resistance-related genes was low in the isolates from Anhui. This finding is consistent with the results for *Salmonella* isolated from Shandong [12] and Henan [11]. There is a certain correlation between the presence of drug-resistance genes in isolates and their drug susceptibility [58]. These resistance-related genes showed higher carrier rates in isolates with corresponding resistance phenotypes, e.g., the carrier rates of *aac(6’)-Ib-cr*, *qnrB*, and *qnrS* were 100% in quinolone-resistant isolates, and *aadA* and *aph(3′)-IIa* were present in 93.33% of isolates that were resistant to aminoglycoside antibiotics. Correlation analysis found that most of the resistance genes in the isolates were significantly positively correlated with their resistance phenotypes. However, the carrying of drug resistance genes is not completely consistent with the drug resistance of the isolate because bacteria have multiple drug resistance mechanisms, such as efflux pumps, drug resistance gene mutations, and biofilms formation [59]. Some isolates also carry resistance-related genes but do not show corresponding resistance phenotypes, which might be related to the selective silencing of some genes under specific conditions [59]. To clarify the mechanism, it is necessary to conduct in-depth research on the biological characteristics and genomic information of the isolates. However, the presence of drug resistance genes in an isolate indicated that it is likely to mutate into the corresponding antibiotic-resistant isolate [60].

*Salmonella* virulence-factor-encoding genes are primarily situated in discrete genomic regions distributed throughout the chromosome, known as *Salmonella* pathogenicity islands (SPIs), which help pathogens evade the host immune system while exerting their pathogenicity [61]. Among them, SPI1, SPI2, SPI3, SPI4, and SPI5 have been studied systematically [62]. In this study, certain virulence genes, including *invA*, *mgtC*, and *stn*, were detected in all *Salmonella* isolates, while *siiE* and *sopB* exhibited notably high carrier rates, suggesting their potential significance in *Salmonella* pathogenesis, which is consistent with previous findings in chickens and ducks reported by Zhang et al. [63] and Yang et al. [64]. The *sseL* gene significantly enhanced the virulence of *S.* Pullorum in chickens and suppressed the activation of cellular inflammatory response [65]. The *spvB* gene, a crucial effector encoded within this locus, is strongly linked to *Salmonella* pathogenicity, for example, by interfering with autophagy and iron homeostasis [66]. Previous investigations identified *spvB* as a potential plasmid-encoded virulence gene in *S.* Pullorum, with detection rates as high as 98.0%. [63], which differed from another study reporting that *spvB* was found in 10% of isolates of *Salmonella* spp. [23]. Another study showed that *spvB* is not present in *S.* Typhimurium [67]. In this study, the positive carrier rate of *sseL* and *spvB* genes showed different distribution patterns among *Salmonella* serovars, which might be closely related to bacterial invasion and the cellular immune response triggered by invasion [68]. These findings indicate that virulence genes are extensively present in *Salmonella* isolates from chickens in Anhui.

Bacterial biofilm is an extracellular matrix composed of polysaccharides, lipids, proteins, and extracellular DNA secreted by bacteria and carbohydrates in the environment, which, as one of the most important antistress mechanisms of bacteria, can endow biofilm bacteria with strong drug resistance and immune escape, resulting in persistent infection [69]. Previous research has shown that biofilm formation by *Salmonella* plays a significant role in its pathogenicity [70] and high potential on common contact surfaces with chicken products [71]. In this study, 52.78% of *Salmonella* isolates could produce a biofilm, most of which were weak biofilm-producing isolates. Further analysis revealed that the proportions of moderate and strong biofilm-producing isolates among *S.* Typhimurium, *S.* Thompson, *S.* Indiana, and *S.* Pullorum were higher than in the other serovars. Similar to our results, 62% of *S.* Enteritidis and 73.8% of *S.* Typhimurium isolates from avian sources exhibited the ability to form biofilms, with *S.* Enteritidis demonstrating a strong capacity for adhesion [72]. Among *Salmonella* isolated from chickens in South Africa, the proportion of isolates producing biofilms at different temperatures reached 86.44–88.14%, and *S.* Heidelberg and *S.* Weltevreden were the serovars with the highest biofilm-forming capacities [73]. Another study found that all of the *Salmonella* serovars were isolated from meat, with 75.86% exhibiting moderate biofilm formation and 24.14% displaying strong biofilm formation. *S.* Enteritidis was identified as the most potent biofilm producer. [74]. Silva et al. [75] believed that *S.* Gallinarum and *S.* Minnesota had stronger biofilm production abilities than the *S.* Enteritidis, *S.* Typhimurium, and *S.* Heidelberg serovars. However, the impact of incubation temperature on biofilm formation was found to be more significant than that of the serovar [72,73]. The results of different studies indicated that the differences in biofilm production ability might be more related to the source, region, and characteristics of the isolates themselves.

MLST has emerged as a fundamental method for bacterial isolate classification into strains, and is being increasingly utilized by both reference and diagnostic laboratories for epidemiological investigations and outbreak studies [76,77]. In this study, sequence analysis revealed ST11, ST19, and ST92 to be the most prevalent sequence types (STs). All 47 *S.* Enteritidis isolates were assigned into ST11, which was consistent with a previous study on *S.* Enteritidis in China spanning from 2011 to 2016 [78]. The results of two independent studies of *Salmonella* isolated from chickens in Shandong were also consistent with our results, finding that ST11 had the highest isolation rates in both breeder farms and free-range flocks [15,79]. In addition, ST11 has been detected in various hosts, including humans, poultry, food sources, and numerous wild animal species, such as reptiles, with a wide geographic distribution spanning Asia, Africa, South America, and Europe [80]. In the present study, ST19 was the most prevalent ST in *S.* Typhimurium isolates, and *S.* Typhimurium isolates also contained ST34 and ST1544. ST19 is very common in *S.* Typhimurium isolated from chicken flocks [25], and is the most common ST isolated from humans and animal-based food products across the world [81,82]. Moreover, ST34 and ST1544 have been identified from human and animal samples in China [83,84]. A study found that ST34 was associated with a higher MDR rate and more complex MDR patterns than ST19 [82]. Although there were only two isolates of ST34 in our study, they were both MDR isolates, which supported the MDR status of ST34 [85].

ST92 contains *S.* Pullorum and *S.* Gallinarum isolates, which are the etiological agents of pullorum disease (PD) and fowl typhoid (FT), respectively, causing huge economic losses to the poultry industry, especially in developing countries [86]. In China, ST92 is widely present in chicken flocks, and was the most common ST in some studies [17,78]. Despite the limited data from this study, we found differences in the prevalence of *Salmonella* STs in different regions of Anhui Province. These results showed that there are a variety of ST *Salmonella* epidemics in chicken flocks in Anhui, and further measures should be taken to prevent *Salmonella* from causing harm to the health of chicken flocks and compromising public health.

## 5. Conclusions

In this study, we explored the prevalence, antimicrobial resistance, and molecular characteristics of *Salmonella* isolated from diseased and clinically healthy chickens in Anhui, China. We found that the dominant *Salmonella* serovars among the isolates were clinically significant *S.* Enteritidis, *S.* Typhimurium, and *S.* Pullorum, and the majority of other isolates were also associated with salmonellosis in animals and humans. The determination of the drug resistance of *Salmonella* isolates and the distribution of drug-resistance-related genes provide basic data for the rational use of antibiotics and monitoring of changes in *Salmonella* drug resistance. The determination of virulence genes and biofilms enriched our knowledge regarding the molecular pathogenic properties of the isolates. MLST analysis showed the prevalence of various ST-type *Salmonella* in Anhui Province, among which ST11, ST19, and ST92 are dominant isolates. This study will aid in the continuous monitoring of the genetic diversity of *Salmonella* isolated from chickens in Anhui and might reveal differences in the epidemiology, evolution, and genetic traits that influence control and treatment.

## Figures and Tables

**Table 1:** Primers for antimicrobial resistance and virulence genes of *Salmonella*.

Location/Function	Gene	Primer Sequence (5′-3′)	Reference
ß-Lactams	*bla_TEM_*	F: CAGCGGTAAGATCCTTGAGA R: ACTCCCCGTCGTGTAGATAA	[20]
	*bla_CMY-2_*	F: TGGCCGTTGCCGTTATCTAC R: CCCGTTTTATGCACCCAT GA	[20]
Aminoglycoside resistance genes	*aadA*	F: ATCCTTCGGCGCGATTTTG R: GCAGCGCAATGACATTCTTG	[21]
	*strA*	F: CCAATCGCAGATAGAAGGC R: CTTGGTGATAACGGCAATTC	[21]
	*aph(3′)-IIa*	F: TCCGGTGCCCTGAATGAACT R: ACG GGT AGC CAA CGC TAT GT	[20]
Quinolone resistance genes	*qnrB*	F: GATCGTGAAAGCCAGAAAGG R: ACGATGCCTGGTAGTTGTCC	[17]
	*qnrS*	F: ACGACATTCGTCAACTGCAA R: TAAATTGGCACCCTGTAGGC	[17]
	*aac(6′)-Ib-cr*	F: TTGCGATGCTCTATGAGTGGCTA R: CTCGAATGCCTGGCGTGTTT	[17]
Tetracycline resistance genes	*tetA*	F: GCGCCTTTCCTTTGGGTTCT R: CCACCCGTTCCACGTTGTTA	[17]
	*tetB*	F: CATTAATAGGCGCATCGCTG R: TGAAGGTCATCGATAGCAGG	[17]
Sulfonamide resistance genes	*sul1*	F: CTTCGATGAGAGCCGGCGGC R: GCAAGGCGGAAACCCGCGCC	[17]
	*sul2*	F: GCGCTCAAGGCAGATGGCATT R: GCGTTTGATACCGGCACCCGT	[17]
Chloramphenicol resistance genes	*cat1*	F: CTTGTCGCCTTGCGTATAAT R: ATCCCAATGGCATCGTAAAG	[21]
	*floR*	F: AACCCGCCCTCTGGATCAAGTCAA R: CAAATCACGGGCCACGCTGTATC	[22]
SPI-1	*invA*	F:CTGGCGGTGGGTTTTGTTGTCTTCTCTATT R:AGTTTCTCCCCCTCTTCATGCGTTACCC	[23]
SPI-2	*sseL*	F: GCCCCTTCCAGATTACTTTATATG R: TGCTTAATATATTTTCTTTGGTGG	[22]
SPI-3	*mgtC*	F: AAAGACAATGGCGTCAACGTATGG R: TTCTTTATAGCCCTGTTCCTGAGC	[22]
SPI-4	*siiE*	F:GGAGTATCGATAAAGATGTT R: GCGCGTAACGTCAGAATCAA	[23]
SPI-5	*sopB*	F:CGGACCGGCCAGCAACAAAACAAGAAG R: TAGTGATGCCCGTTATGCGTGAGTGTATT	[23]
*Salmonella* plasmid virulence	*spvB*	F:CTATCAGCCCCGCACGGAGAGCAGTTTT R: GGAGGAGGCGGTGGCGGTGGCATCATA	[23]
*Salmonella* enterotoxin	*stn*	F: AGCGTTCAGGTACAGATTCAACA R: AAATTCGTAACCCGCTCTCGT	[22]

**Table 2:** Prevalence of *Salmonella* isolates among type and number of samples.

Sample Type	Number of Samples	Number of Isolates	Isolation Rate (%)
Cloacal swab	1500	51	3.40
Pathological tissue	408	57	13.97
Total	1908	108	5.66

**Table 3:** Antimicrobial resistance of *Salmonella* isolated from chickens.

Classes	Antimicrobials	Concentrations(µg)	Number of Isolates	Resistance (%)
Penicillin	AMP	10	66	61.11
β-lactams	AMC	20/10	24	22.22
Cephems	CRO	30	36	33.33
	CTX	30	30	27.78
	CN	30	36	33.33
Aminoglycosides	GEN	10	12	11.11
	AMK	30	4	3.70
	NEO	30	7	6.48
Tetracyclines	TET	30	51	47.22
	DOX	30	49	45.37
Quinolones	CIP	5	6	5.56
	ENR	10	2	1.85
	LEV	5	8	7.41
	NOR	10	6	5.56
Chloramphenicols	CHL	30	24	22.22
	FLO	30	24	22.22
Sulfonamides	SXT	23.75/1.25	42	48.89
	TMP	5	42	48.89
Macrolides	AZM	15	14	12.96
Nitrofurans	FUR	100	20	18.52
Carbapenems	IPM	10	0	0
Polypeptide	PB	300	0	0
Fosfomycin	FOS	200	4	3.70
Monobactams	AZT	30	37	34.26

**Table 4:** Genotypic drug resistance characteristics of *Salmonella* Isolates.

Antimicrobials	Gene	No. (%)
ß-Lactamase (*n* = 66)	*blaTEM*	55 (83.33)
	*blaCMY-2*	59 (89.39)
Quinolones (*n* = 9)	*aac(6′)-Ib-cr*	9 (100.00)
	*qnrB*	9 (100.00)
	*qnrS*	9 (100.00)
Aminoglycoside (*n* = 15)	*aadA1*	14 (93.33)
	*strA*	5 (33.33)
	*aph(3′)-IIa*	14 (93.33)
Tetracycline (*n* = 52)	*tetA*	42 (80.77)
	*tetB*	22 (42.31)
Sulfonamide (*n* = 51)	*sul1*	32 (62.75)
	*sul2*	31 (60.78)
Chloramphenicol (*n* = 24)	*catA1*	17 (70.83)
	*floR*	23 (95.83)

## Data Availability

All data generated or analyzed during this study are included in this published article (and its Supplementary Information Files).

## Supplementary Materials

The following supporting information can be downloaded at: https://www.mdpi.com/article/10.3390/pathogens12030465/s1, Figure S1: Correlation analysis among different classes of antimicrobials resistance phenotypes of *Salmonella* isolates in Anhui, * *p* < 0.05, ** *p* < 0.01, correlation analysis by pearson method; Table S1: *Salmonella* isolation results from cloacal swab samples from 50 chicken farms; Table S2: Prevalence of *Salmonella* isolated from chicken in Anhui; Table S3: Antimicrobial resistance spectrum of the *Salmonella* isolates.
