# Supplementary material for: Prevalence and Characterization of Salmonella Isolated from Chickens in Anhui, China
Source: Pathogens. 2023 Mar 16;12(3):465. doi: 10.3390/pathogens12030465 (PMC10054756; doi:10.3390/pathogens12030465)
Supplement: Supplementary file 1 [file pathogens-12-00465-s001.zip › Supplementary Tables .pdf]

Table S1. *Salmonella* isolation results from cloacal swab samples from 50 chicken farms

Number	Farm scale [a]	Breeding mode	farm type	source area	*Salmonella* isolation results
1	large	cage	laying hen	Dingyuan	0/30
2	medium	free-range	broiler	Hefei	5/30
3	medium	cage	laying hen	Hefei	0/30
4	large	cage	laying hen	Feixi	0/30
5	large	cage	laying hen	Hexian	0/30
6	medium	cage	laying hen	Fuyang	0/30
7	medium	cage	laying hen	Fanchang	2/30
8	medium	cage	laying hen	Liu'an	0/30
9	medium	cage	laying hen	Hefei	7/30
10	large	cage	laying hen	Hefei	0/30
11	small	free-range	broiler	Wuhu	5/30
12	large	cage	laying hen	Wuhu	4/30
13	medium	cage	laying hen	Wuhu	0/30
14	medium	cage	laying hen	Chaohu	0/30
15	medium	cage	laying hen	Guzhen	5/30
16	large	cage	laying hen	Guzhen	0/30
17	large	cage	laying hen	Changfeng	0/30
18	large	cage	laying hen	Changfeng	0/30
19	medium	cage	laying hen	Changfeng	0/30
20	small	free-range	laying hen	Fengtai	0/30
21	medium	free-range	broiler	Huainan	0/30
22	large	cage	laying hen	Huainan	3/30
23	large	cage	broiler	Huainan	0/30
24	medium	cage	laying hen	Feidong	0/30
25	medium	cage	laying hen	Feidong	0/30
26	medium	cage	laying hen	Heifei	2/30
27	large	cage	laying hen	Xuancheng	0/30
28	medium	cage	laying hen	Xuancheng	0/30
29	medium	cage	laying hen	Fuyang	0/30
30	large	cage	laying hen	Fuyang	0/30
31	large	cage	laying hen	Fuyang	2/30
32	medium	cage	laying hen	Dingyuan	0/30
33	medium	cage	laying hen	Dingyuan	0/30
34	large	cage	laying hen	Wuhu	0/30
35	medium	cage	laying hen	Wuhu	0/30
36	large	cage	laying hen	Wuhu	0/30
37	medium	cage	laying hen	Fanchang	0/30
38	medium	cage	laying hen	Hanshan	0/30
39	large	cage	laying hen	Hanshan	0/30
40	large	cage	laying hen	Liu'an	0/30
41	medium	cage	laying hen	Shouxian	3/30

42	medium	cage	laying hen	Huoqiu	0/30
43	medium	cage	laying hen	Liu'an	5/30
44	medium	cage	laying hen	Feidong	0/30
45	medium	cage	laying hen	Feidong	3/30
46	medium	cage	laying hen	Feixi	1/30
47	small	free-range	broiler	Feixi	2/30
48	medium	cage	laying hen	Hefei	2/30
49	large	cage	laying hen	Changfeng	0/30
50	large	cage	laying hen	Sixian	0/30

[a] Large-scale farm with a breeding scale ≥ 20,000 chickens, medium- scale farm with a breeding scale ≥5,000 and < 20,000 chickens, small-scale farm with a breeding scale <5,000 chickens.

Table S2. Prevalence of *Salmonella* isolated from chicken in Anhui

NO.	*Salmonella* isolate number	Sample type	Breeding mode	Chicken type	Sample source region
1	SCF1	pathological tissue	cage	laying hen	Changfeng
2	SHF1	cloacal swab	cage	laying hen	Hefei
3	SHF2	cloacal swab	cage	laying hen	Hefei
4	SFD1	pathological tissue	free-range	broiler	Feidong
5	SFD2	pathological tissue	free-range	broiler	Feidong
6	SWH1	cloacal swab	cage	laying hen	Wuhu
7	SWH2	cloacal swab	cage	laying hen	Wuhu
8	SWH3	cloacal swab	cage	laying hen	Wuhu
9	SWH4	cloacal swab	cage	laying hen	Wuhu
10	SFX1	pathological tissue	free-range	broiler	Feixi
11	SHF3	pathological tissue	cage	laying hen	Hefei
12	SCF2	pathological tissue	cage	laying hen	Changfeng
13	SGZ1	pathological tissue	free-range	broiler	Guzhen
14	SAQ1	pathological tissue	cage	laying hen	Anqing
15	SAQ2	pathological tissue	cage	laying hen	Anqing
16	SHQ1	pathological tissue	free-range	broiler	Huoqiu
17	SHN1	pathological tissue	cage	laying hen	Huainan
18	SHN2	pathological tissue	cage	laying hen	Huainan
19	SFX2	pathological tissue	cage	laying hen	Feixi
20	SSX1	pathological tissue	cage	laying hen	Shouxian
21	SHF4	pathological tissue	cage	laying hen	Hefei
22	SHF5	pathological tissue	free-range	broiler	Hefei
23	SHX1	pathological tissue	free-range	broiler	Hexian
24	SFD3	pathological tissue	free-range	broiler	Feidong
25	SFD4	pathological tissue	cage	laying hen	Feidong
26	SFX3	pathological tissue	free-range	broiler	Feixi
27	SFD5	cloacal swab	cage	laying hen	Feidong
28	SFD6	cloacal swab	cage	laying hen	Feidong

29	SFD7	cloacal swab	cage	laying hen	Feidong
30	SHF6	cloacal swab	cage	laying hen	Hefei
31	SHF7	cloacal swab	cage	laying hen	Hefei
32	SHF8	cloacal swab	cage	laying hen	Hefei
33	SHF9	cloacal swab	cage	laying hen	Hefei
34	SHF10	cloacal swab	cage	laying hen	Hefei
35	SHF11	cloacal swab	cage	laying hen	Hefei
36	SHF12	cloacal swab	cage	laying hen	Hefei
37	SHF13	pathological tissue	free-range	broiler	Hefei
38	SHF14	pathological tissue	cage	laying hen	Hefei
39	SHF15	pathological tissue	free-range	broiler	Hefei
40	SFD8	pathological tissue	cage	laying hen	Feidong
41	SCF3	pathological tissue	cage	laying hen	Changfeng
42	SCF4	pathological tissue	cage	laying hen	Changfeng
43	SFD9	pathological tissue	free-range	broiler	Feidong
44	SFD10	pathological tissue	free-range	broiler	Feidong
45	SCH1	pathological tissue	cage	laying hen	Chaohu
46	SDY1	pathological tissue	free-range	laying hen	Dingyuan
47	SHF16	cloacal swab	free-range	broiler	Hefei
48	SHF17	cloacal swab	free-range	broiler	Hefei
49	SHF18	cloacal swab	free-range	broiler	Hefei
50	SHF19	cloacal swab	free-range	broiler	Hefei
51	SHF20	cloacal swab	free-range	broiler	Hefei
52	SHF21	pathological tissue	cage	laying hen	Hefei
53	SHF22	pathological tissue	cage	laying hen	Hefei
54	SHF23	pathological tissue	cage	laying hen	Hefei
55	SCF5	pathological tissue	free-range	broiler	Changfeng
56	SFD11	pathological tissue	free-range	broiler	Feidong
57	SFC1	pathological tissue	cage	laying hen	Fangchang
58	SHF24	cloacal swab	cage	laying hen	Hefei
59	SHF25	cloacal swab	cage	laying hen	Hefei
60	SHF26	pathological tissue	free-range	broiler	Hefei
61	SLA1	pathological tissue	cage	laying hen	Liu'an
62	SFY1	pathological tissue	cage	laying hen	Fuyang
63	SCF6	pathological tissue	cage	laying hen	Changfeng
64	SLA2	pathological tissue	cage	laying hen	Liu'an
65	SCF7	pathological tissue	free-range	broiler	Changfeng
66	SCH2	pathological tissue	cage	laying hen	Chaohu
67	SHF27	pathological tissue	cage	laying hen	Hefei
68	SCH3	pathological tissue	cage	laying hen	Chaohu
69	SCH4	pathological tissue	cage	laying hen	Chaohu
70	SCH5	pathological tissue	cage	laying hen	Chaohu
71	SFC2	cloacal swab	cage	laying hen	Fangchang
72	SFC3	cloacal swab	cage	laying hen	Fangchang

73	SSX2	cloacal swab	cage	laying hen	Shouxian
74	SSX3	cloacal swab	cage	laying hen	Shouxian
75	SSX4	cloacal swab	cage	laying hen	Shouxian
76	SLA3	pathological tissue	cage	laying hen	Liu’an
77	SLA4	pathological tissue	cage	laying hen	Liu’an
78	SLA5	pathological tissue	cage	laying hen	Liu’an
79	SLA6	cloacal swab	cage	laying hen	Liu’an
80	SLA7	cloacal swab	cage	laying hen	Liu’an
81	SLA8	cloacal swab	cage	laying hen	Liu’an
82	SLA9	cloacal swab	cage	laying hen	Liu’an
83	SLA10	cloacal swab	cage	laying hen	Liu’an
84	SHQ2	pathological tissue	cage	laying hen	Huoqiu
85	SHQ3	pathological tissue	free-range	broiler	Huoqiu
86	SHN3	cloacal swab	cage	laying hen	Huainan
87	SHN4	cloacal swab	cage	laying hen	Huainan
88	SHN5	cloacal swab	cage	laying hen	Huainan
89	SHN6	pathological tissue	free-range	broiler	Huainan
90	SHN7	pathological tissue	free-range	broiler	Huainan
91	SGZ2	cloacal swab	cage	laying hen	Guzhen
92	SGZ3	cloacal swab	cage	laying hen	Guzhen
93	SGZ4	cloacal swab	cage	laying hen	Guzhen
94	SGZ5	cloacal swab	cage	laying hen	Guzhen
95	SGZ6	cloacal swab	cage	laying hen	Guzhen
96	SFY2	cloacal swab	cage	laying hen	Fuyang
97	SFY3	cloacal swab	cage	laying hen	Fuyang
98	SFX4	cloacal swab	cage	laying hen	Feixi
99	SFX5	pathological tissue	cage	laying hen	Feixi
100	SFX6	pathological tissue	cage	laying hen	Feixi
101	SFX7	pathological tissue	free-range	broiler	Feixi
102	SFX8	cloacal swab	cage	laying hen	Feixi
103	SFX9	cloacal swab	cage	laying hen	Feixi
104	SWH5	cloacal swab	free-range	broiler	Wuhu
105	SWH6	cloacal swab	free-range	broiler	Wuhu
106	SWH7	cloacal swab	free-range	broiler	Wuhu
107	SWH8	cloacal swab	free-range	broiler	Wuhu
108	SWH9	cloacal swab	free-range	broiler	Wuhu

Table S3. Antimicrobials resistance spectrum of the isolates

**No.of antibacterials**	**Drug resistant spectrum**	**No.of Strains**	**Drug resistance rate/%**
0		20	18.52
1	TMP	2	1.85
1	LEV	1	0.93
1	TMP	5	4.63
1	AZM	2	1.85
1	FUR	14	12.93
2	AMP+ FUR	1	0.93
2	AMP+TMP	1	0.93
3	AMP+TET+DOX	11	10.19
	AMP+STX+TMP	4	3.70
4	AMP+STX+TMP+ FUR	2	1.85
	AMP+TET+DOX+ AZT	1	0.93
	AMP+TET+DOX+ FUR	1	0.93
	AMP+TET+DOX+ TMP	1	0.93
	AMP+AMC+TET+DOX	2	1.85
6	AMP+ CRO +CN+TET+STX+ AZT	3	2.78
7	AMP+ CTX+ TET + DOX +CHL+FLO+ TMP	2	1.85
	AMP+ CRO +CN+TET+ DOX+STX+ AZT	3	2.78
8	AMP+ CTX+ TET+DOX +CHL+FLO+ STX+TMP	1	0.93
	AMP+ CRO +CN+NEO+TET+ DOX+STX+ AZT	1	0.93
	AMP+ CRO +CTX+CN+AMK+CHL+FLO+ AZT	2	1.85
	AMP+AMC+ CRO +CN+ TET+DOX+ STX+ AZT	2	1.85
11	AMP+AMC+ CRO+ CTX+CN+ CHL+FLO+ STX	2	1.85
	+TMP+AZM+ AZT	1	0.93
	AMP+ CEN+NEO+ TET + DOX +CIP+NOR+	3	2.78
	CHL+FLO+STX +TMP	2	1.85
	AMP+ CRO+ CTX+CN+ CEN+ TET + DOX+ STX		
	+TMP+AZM+ AZT		
	AMP+ CRO+ CTX+CN+ TET + DOX+ CHL+FLO+STX		
	+TMP+ AZT		
12	AMP+AMC+ CRO+ CTX+CN+ TET+DOX+TMP+	9	8.33
	CHL+FLO+STX +TMP+ AZT	1	0.93
	AMP+AMC+ CRO+ CTX+CN+ CEN+ TET + DOX+ STX		
	+TMP+AZM+ AZT		
13	AMP+AMC+ CRO+ CTX+CN+ CEN+ TET+DOX+LEV+		
	STX +TMP+AZM+ AZT	2	1.85
	AMP+AMC+ CRO+ CTX+CN+ TET+DOX+	1	0.93
	CHL+FLO+STX +TMP+ FUR+ AZT		
18	AMP+AMC+ CRO+ CTX+CN+ CEN+		

	TET+DOX+CIP+ENR+LEV+NOR+ STX +TMP+ FOS+ AZT	1	0.93
19	AMP+AMC+ CRO+ CTX+CN+ GEN+AMK+NEO+DOX+CIP+ENR+LEV + NOR+ CHL+FLO+ STX +TMP+ AZM+ FOS + AZT	1	0.93
	AMP+AMC+ CRO+ CTX+CN+ GEN+NEO+ TET+DOX+CIP+ LEV+NOR+ CHL+FLO+STX+ TMP+ AZM+ FOS + AZT	2	1.85
20	AMP+AMC+ CRO+ CTX+CN+ GEN+AMK+ NEO+ TET+DOX+CIP+ ENR+LEV+NOR+ CHL+ FLO+ STX +TMP+ FUR+ AZT	1	0.93
