# Supplementary figures and images for: Prevalence and Characterization of Salmonella Isolated from Chickens in Anhui, China
Source: Pathogens. 2023 Mar 16;12(3):465. doi: 10.3390/pathogens12030465 (PMC10054756; doi:10.3390/pathogens12030465)

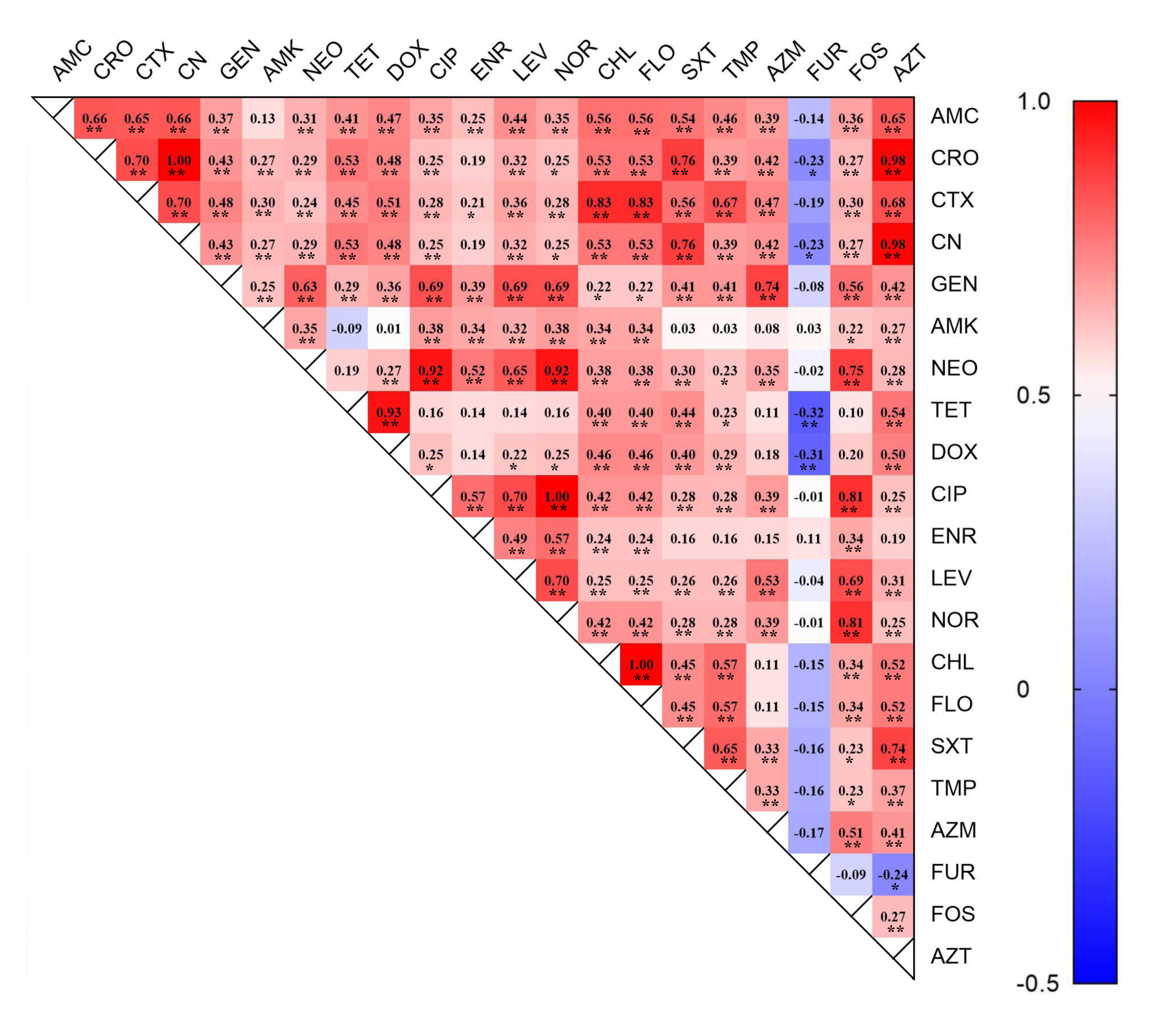

Supplement: Supplementary file 1 [file pathogens-12-00465-s001.zip › Figure S1.tif]
